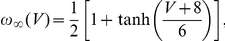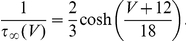# Synchronous Bursts on Scale-Free Neuronal Networks with Attractive and Repulsive Coupling

**DOI:** 10.1371/journal.pone.0015851

**Published:** 2011-01-06

**Authors:** Qingyun Wang, Guanrong Chen, Matjaž Perc

**Affiliations:** 1 Department of Dynamics and Control, Beihang University, Beijing, China; 2 Department of Electronic Engineering, City University of Hong Kong, Hong Kong SAR, China; 3 Department of Physics, Faculty of Natural Sciences and Mathematics, University of Maribor, Maribor, Slovenia; German Cancer Research Center, Germany

## Abstract

This paper investigates the dependence of synchronization transitions of bursting oscillations on the information transmission delay over scale-free neuronal networks with attractive and repulsive coupling. It is shown that for both types of coupling, the delay always plays a subtle role in either promoting or impairing synchronization. In particular, depending on the inherent oscillation period of individual neurons, regions of irregular and regular propagating excitatory fronts appear intermittently as the delay increases. These delay-induced synchronization transitions are manifested as well-expressed minima in the measure for spatiotemporal synchrony. For attractive coupling, the minima appear at every integer multiple of the average oscillation period, while for the repulsive coupling, they appear at every odd multiple of the half of the average oscillation period. The obtained results are robust to the variations of the dynamics of individual neurons, the system size, and the neuronal firing type. Hence, they can be used to characterize attractively or repulsively coupled scale-free neuronal networks with delays.

## Introduction

It is well known that synchronization in neuronal networks is particularly relevant for the efficient processing and transmission of information (see *e.g.* [1], [2]). Experiments have shown that synchronized states can occur in many special areas of the brain, such as the olfactory system or the hippocampal region [3]–[5]. By using functional magnetic resonance imaging (fMRI) to record brain activity from both speakers and listeners during natural verbal communication, a recent study has shown that speaker-listener neural coupling underlies successful communication by means of synchronization [6]. Theoretically, neuronal synchronization on complex networks has been explored in detail [7]–[13], leading to several insights that have applicability on real problems in neuroscience. For example, synchronization of gap-junction-coupled neurons has been investigated [11], and by means of the phase resetting curve, phase locking and synchronization in neuronal networks have been investigated as well [14], [15]. Moreover, noise-induced and noise-enhanced synchronization have also been reported in realistic neuronal systems [16], [17]. Interestingly, it was reported that chemical and electrical synapses perform complementary roles in the synchronization of interneuronal networks [18]. Indeed, synchronization, information transmission and signal sensitivity on complex networks are currently hot topics in theoretical neuroscience [19], [20], as evidenced by several recent studies that are devoted to the explorations of this subject [21]–[32].

Previous research highlighted that information transmission delays are inherent to the nervous system because of the finite speed at which action potentials propagate across neuron axons, as well as due to time lapses occurring by both dendritic and synaptic processing [33]. It has been reported, for example, that the beta frequency is able to synchronize over long conduction delays, which corresponds to signals traveling a significant distance in the brain [34]. Thus far, it has also been reported that different time delay lengths can change both qualitative as well as quantitative properties of the dynamics [35]. For example, delays can introduce or destroy stable oscillations, enhance or suppress synchronization, as well as generate complex spatiotemporal patterns on regular neuronal networks. It has also been suggested that time delays can facilitate neural synchronization and lead to many interesting and even unexpected phenomena [36], [37], including zigzag fronts of excitations, clustering antiphase synchronization and in-phase synchronization [38]. Most recently, the synchronizability threshold for an arbitrary network incorporating delays and noise has been derived, and additionally, by means of the scaling theory of the underlying fluctuations, the absolute limit of synchronization efficiency in a noisy environment with uniform time delays has been established [39].

Both phase-attractive (which can be related to excitatory synapses) and phase-repulsive (which can be related to inhibitory synapses) coupling exists in realistic neuronal systems. Hence, it is important to take this explicitly into account in theoretical studies. Effects of phase-repulsive coupling on neuronal dynamics have also been investigated in the past [40]–[42], where such coupling was considered to be related to inhibitory synapses. For example, it has been shown that a pair of excitable FitzHugh-Nagumo neurons can exhibit various firing patterns including multistability and chaotic firing when elements interact phase-repulsively [40]. Moreover, the synchronization of nonidentical dynamical units that are coupled attractively in a small-world network can be improved significantly by the introduction of just a small fraction of phase-repulsive couplings [42]. Dynamics of propagation in coupled neuronal networks with excitatory and inhibitory synapses has been investigated in detail by means of integrate-and-fire neurons [43], [44]. By analyzing a canard mechanism, it has also been shown that synaptic coupling can synchronize neurons at low firing frequencies [45]. However, synchronization on scale-free neuronal networks with phase-repulsive coupling and delay has not yet been investigated.

Here, we aim to extend the scope of research by studying the dependence of synchronization transitions on the information transmission delay over scale-free neuronal networks with attractive or repulsive coupling, respectively. Since a power law distribution of the degree of neurons has been found applicable for the coherence among activated voxels using functional magnetic resonance imaging [46], and moreover, the robustness against simulated lesions of anatomic cortical networks was also found to be most similar to that of a scale-free network [47], our study addresses a relevant system setup which is still amenable to new research. We report several non-trivial effects induced by finite (non-zero) delay lengths, foremost the ability of its fine-tuning towards highly synchronized fronts of excitations. We find that the delay-induced synchronization transitions manifest as well-expressed minima in the measure for spatiotemporal synchrony. Depending on the type of coupling, however, these minima appear every integer multiple of the average oscillation period of bursting oscillations in case of attractive coupling, or they appear every odd multiple of the half of the average oscillation period for repulsive coupling. The results are robust to variations of neuronal dynamics and system size, and appear to be primarily due to the emergence of phase locking between the delay and the time scales, which are inherent to each individual neuron constituting the scale-free network.

## Results

**Figure 1:**
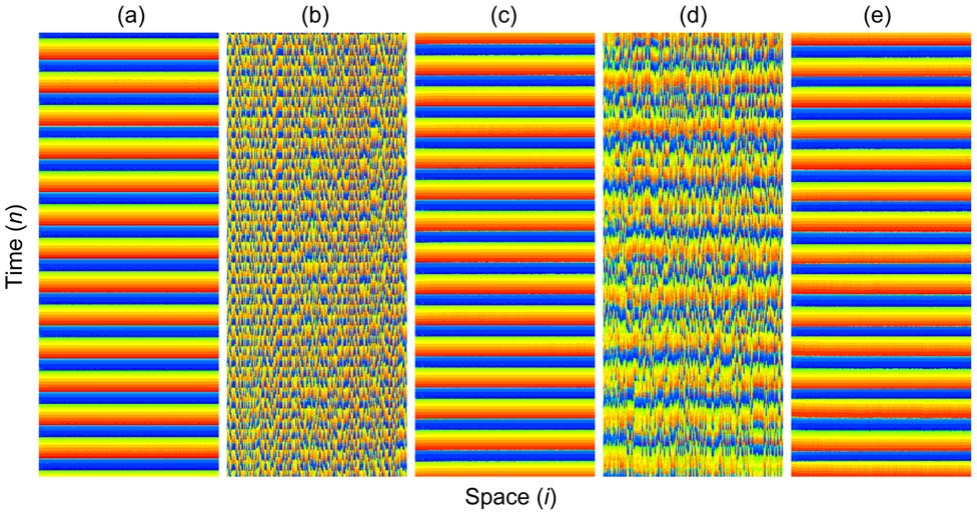
Characteristic space-time plots of the fast variable 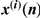 for different information transmission delays 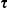. From left to right the delay length is: (a) 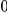, (b) 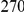, (c) 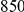, (d) 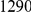 and (e) 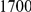. Notice the emergence of complete synchrony in panels (a), (c) and (e). The color coding is linear, red and blue depicting 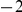 and 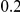 values of 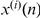, respectively. Other system parameters are: 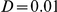, 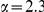 and 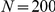.

Firstly, we present in Fig. 1 space-time plots to have a look at characteristic synchronization transitions that can be induced by different information transmission delays. To do so, we employ attractive coupling as a case of example, but note that qualitatively identical space-time plots can be obtained also for repulsive coupling. We set 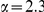, for which individual neurons exhibit simple single-burst excitations. Results presented in Fig. 1(a) indicate that the spatiotemporal dynamics is synchronous if 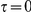, which can be attributed to sufficiently strong attractive coupling. However, if the information transmission delay is increased to 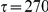 the synchrony deteriorates rather drastically, as can be observed in Fig. 1(b). Interestingly, synchronization seems again fully restored at 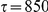, as depicted in Fig. 1(c), but then again disappears for 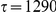 and reappears for 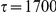, as shown in Figs. 1(d) and (e), respectively. Indeed, we find that such a succession repeats itself for higher values of 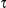, from which we conclude that the information transmission delay can either promote or impair synchronization of neuronal activity on scale-free networks. If inspecting the values of 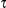 warranting near-perfect synchronization closely, we can observe that they equal roughly integer multiples of 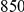, which hints towards an underlying mechanism that can explain our observations.

**Figure 2:**
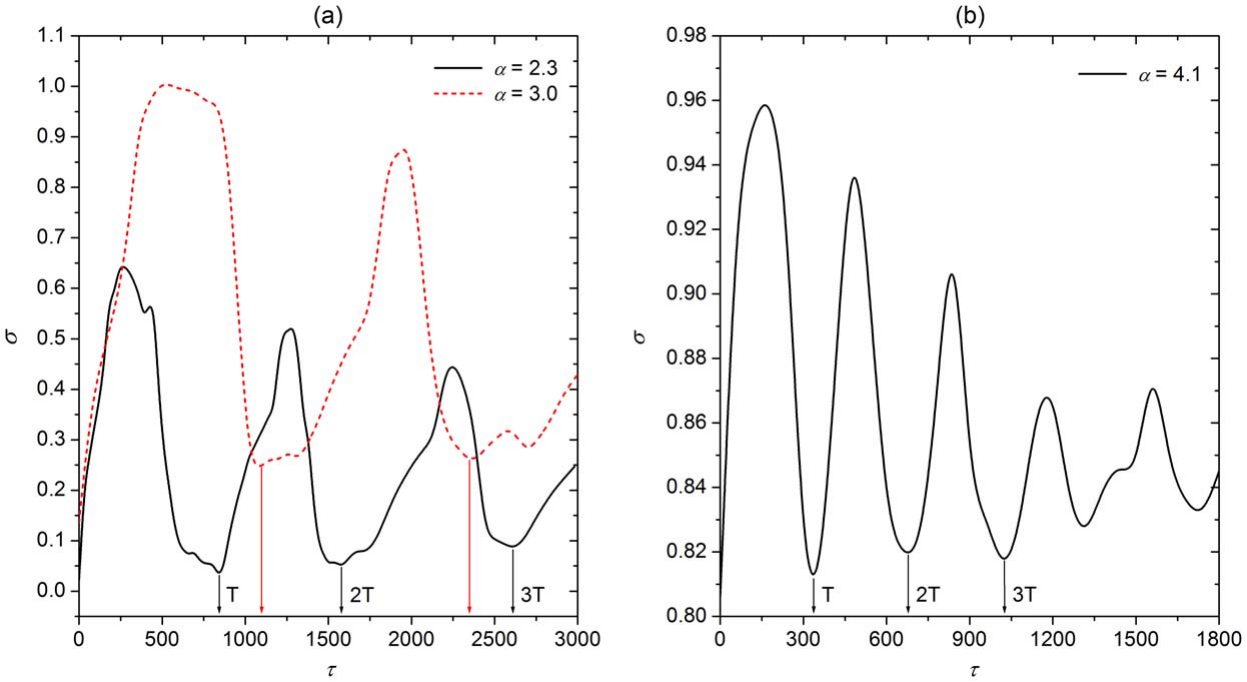
Quantification of synchronization for attractive coupling. Dependence of the synchronization parameter 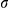 on 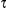 for different values of 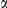, as denoted in the corresponding panels. The undulations of 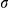 are clearly visible and persist irrespective of 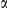. While the minima shift for different 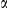, they always occur at integer multiples 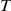, 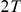, 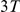 of the average oscillation period of bursting oscillations, as denoted by the vertical arrows.

In order to investigate the impact of different values of 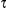 quantitatively, and separately for attractive and repulsive coupling, we calculate the synchronization parameter 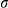 as defined by Eq. (3). Results presented in Figs. 2(a) and (b) were obtained for attractive coupling and three different values of 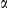. It can be observed that certain values of 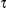 significantly facilitate spatiotemporal synchronization of excitatory fronts on neuronal scale-free networks. In particular, the three minima of 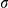 appear at 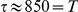, 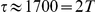 and 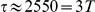 if 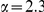. For 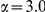, we can observe two minima of 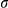 appearing at 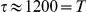 and 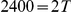. Furthermore, several more minima can be observed for 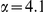 within the considered span of information transmission delays, as depicted in Fig. 2(b). Again it is clear that they appear at integer multiples of the first minimum. This confirms the fact that delay-induced transitions to spatiotemporally synchronized neuronal activity appear intermittently, at integer multiples of a given value of 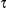. On the other hand, values of 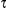 outside these regions impair synchronization significantly, as can be inferred from the rather sharp ascends towards larger values of 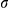 beyond the optimal delays.

**Figure 3:**
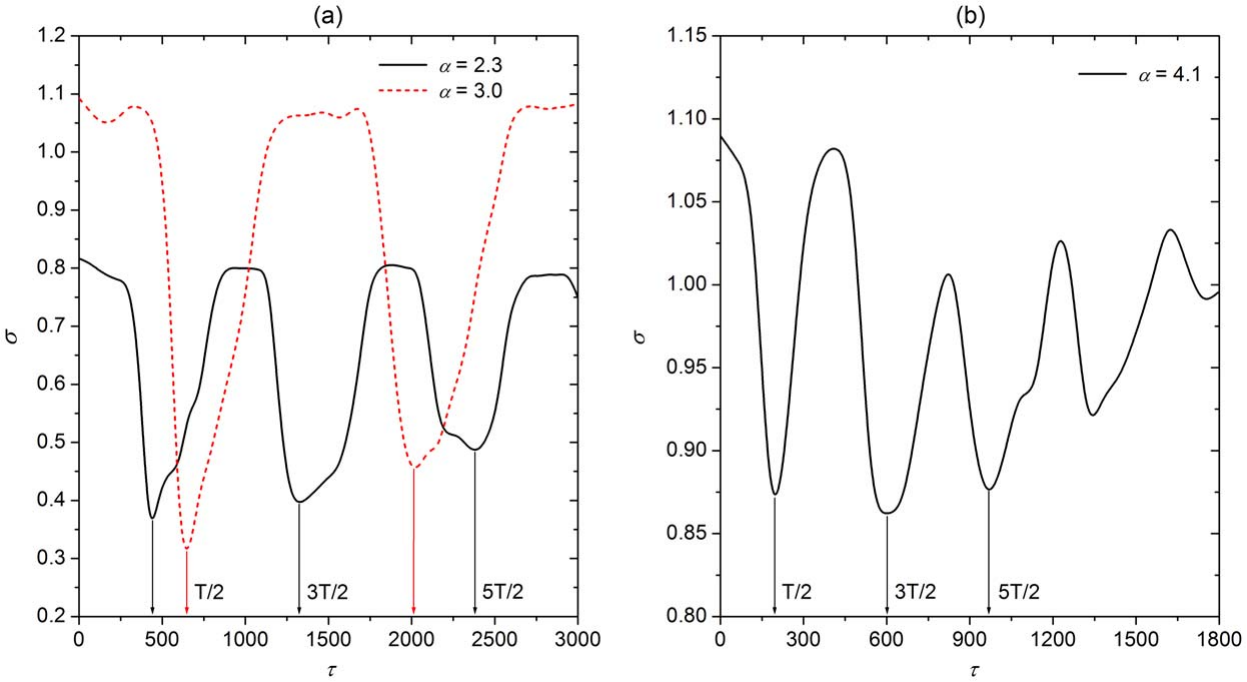
Quantification of synchronization for repulsive coupling. Dependence of the synchronization parameter 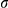 on 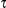 for different values of 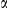, as denoted in the corresponding panels. The undulations of 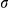 are clearly visible and persist irrespective of 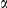. While the minima shift for different 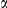, they always occur at odd integer multiples 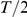, 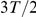, 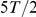 of the half of the average oscillation period of bursting oscillations, as denoted by the vertical arrows.

Performing the same analysis for repulsive coupling reveals several similarities, but also significant differences. Results presented in Figs. 3(a) and (b) indeed have a qualitatively identical outlay with the minima of 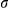 appearing intermittently as 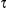 increases, yet the precise values warranting optimal neuronal synchrony are different if compared to the case of attractive coupling. Specifically, the three minima of 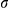 appear at 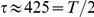, 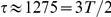 and 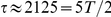 if 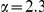, while for 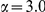 and 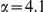 we can observe similar variations with odd integer multiples of half of 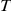 constituting optimal information transmission delays where 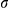 is minimal. As for attractive coupling, values of 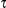 outside these bounds impair synchronization significantly and fast. Altogether, results presented in Figs. 2 and 3 indicate that simple scaling laws account for the description of optimal information transmission delays that warrant near-perfect synchronization of neuronal activity on scale-free networks. While for attractive coupling integer multiples of a given constant period are optimal, for repulsive coupling odd integer multiples of half of the same period have the best effect. Irrespective of the coupling type, delays outside the narrow optimal span impair synchronization significantly.

**Figure 4:**
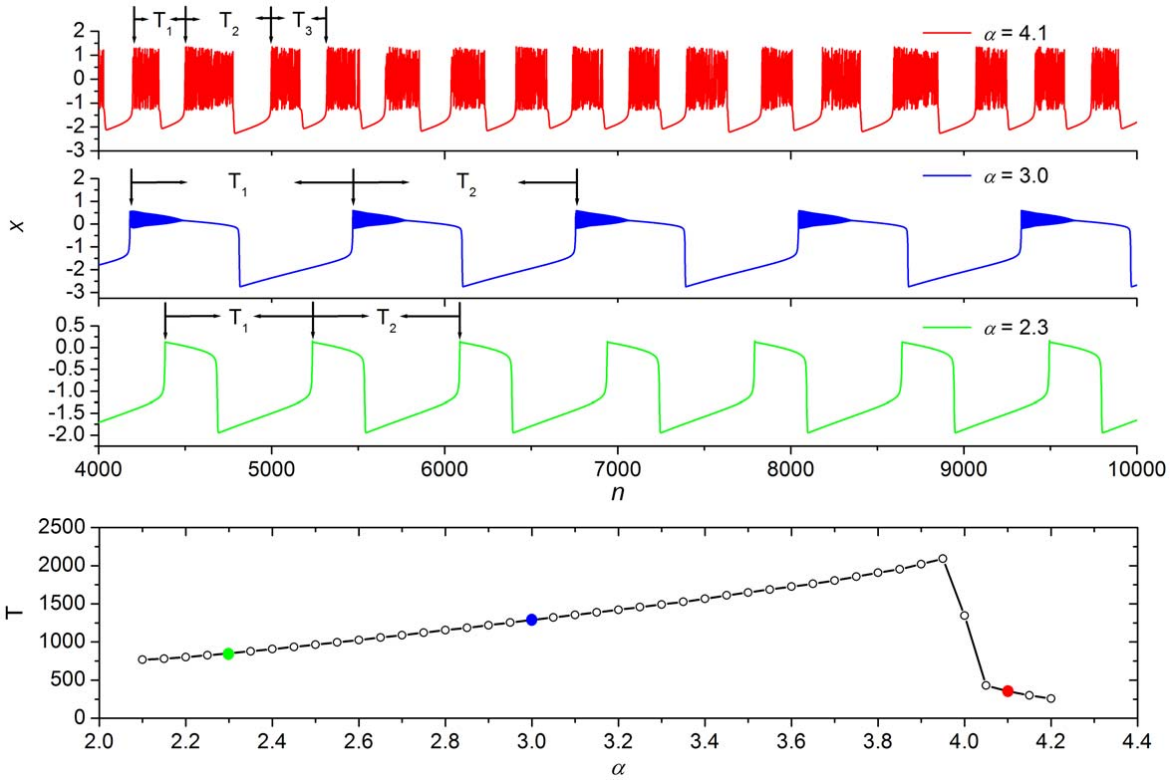
Time series of the Rulkov map for different values of 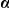 and the determination of the average oscillation period of bursting. *Top three panels:* From top to bottom we have 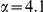, 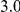 and 2.3, respectively. Evidently, the time between consecutive bursts changes significantly, as denoted by 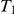, 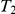 and 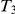, respectively. Simultaneously, different values of 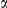 also affect the oscillation period. This gives vital clues as to the location of minima of the synchronization parameter 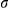 depicted in Figs. 2 and 3. *Bottom panel:* Average oscillation period of bursting oscillations 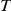 in dependence on 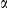, determined as the average over 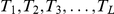. Here 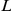 is the total number of periods considered, which was selected large enough to ensure convergence.

It is next of interest to explore and determine the mechanisms behind these observations. We will do this by means of the duration of bursting periods of individual neurons constituting the scale-free network. The top three panels of Fig. 4 depict time courses of the membrane potential 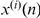 for the values of 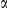 we have used in Figs. 2 and 3 above. It can be observed that, depending on 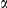, the duration of bursts within a given trace may vary (chaotic bursting [48]), but also that the duration of bursts changes due to different 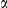 values. This is highlighted by labels 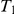, 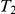 and 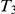 (where applicable) in the top three panels of Fig. 4. From this it is straightforward to determine the average oscillation period of bursting 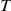 for each particular value of 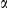, simply as the average over a large enough ensemble 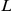 as 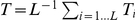. The bottom panel of Fig. 4 shows how the average period 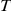 varies with 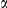. It can be observed that upon exceeding the Hopf bifurcation at 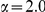 the period 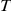 increases fairly linearly, but then drops rather sharply when 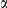 exceeds 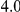.

Upon connecting the values of 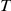 with the optimal information transmission delays observed in Figs. 2 and 3 for the corresponding values of 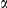, we can establish a good understanding of the mechanism behind the observed synchronization transitions for attractive as well as for repulsive coupling. In particular, from results presented in Fig. 4 it follows that if 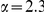 then 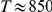, which is exactly the value of 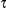 corresponding to the first minimum of 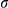 for attractive coupling. Conversely, one half and three times one half of 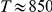 correspond to the first and second minima of 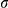 if 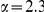 and the coupling is repulsive. For the other two considered values of 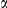, namely 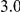 and 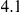, an identical linkage can be established easily from the results presented in Figs. 2 and 3, depending on the type of coupling one is interested in, and the bottom panel of Fig. 4. Apparently, the average period of individual bursts determines the optimal information transmission delay that warrants the best synchrony, *i.e.* minimal 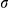, of neuronal firings on the scale-free network. We therefore conclude that for attractive coupling the delay-induced transitions to spatiotemporal synchronization of neuronal activity are due to the locking between 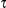 and the average oscillation period of individual neurons constituting the scale-free network. Importantly, because the repulsive coupling can pull adjacent neurons into antiphase synchronization, the optimal delay warranting best synchronization is not equal to full integer multiples of 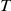. Thus, it is exactly odd integer multiples of one half of the average oscillation period of an individual neuron, where the phase locking between antiphased bursts occurs.

**Figure 5:**
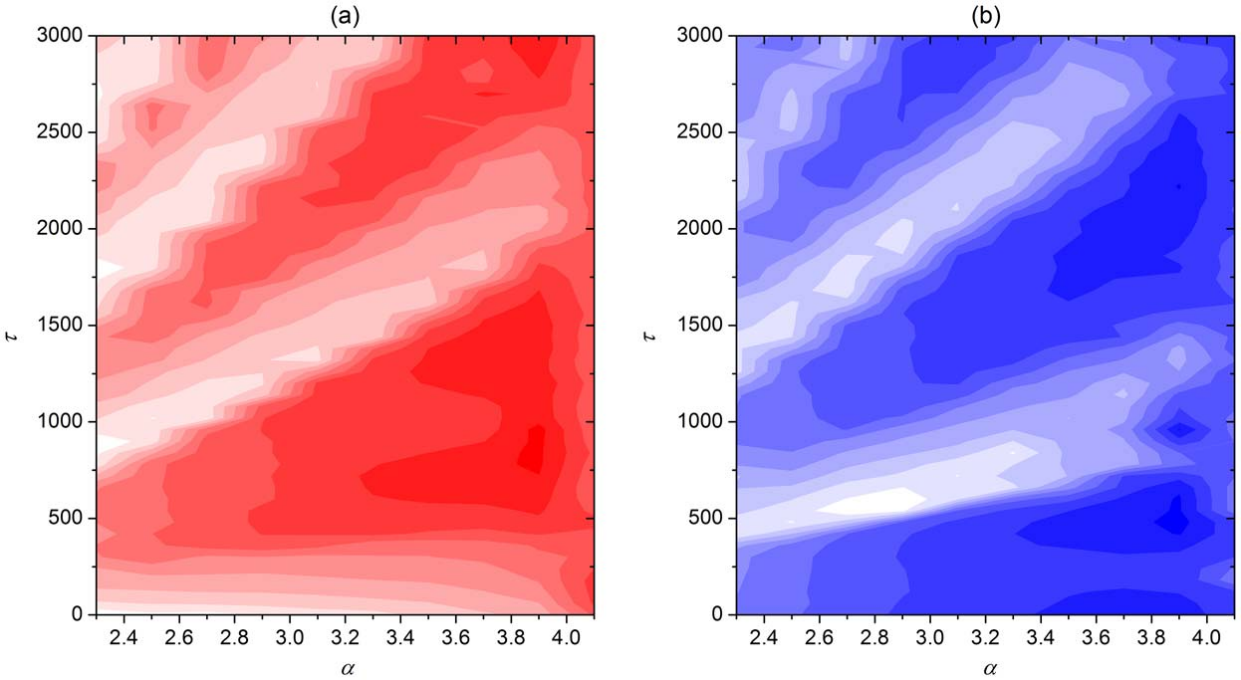
Two-parameter dependence of synchronization among neurons. Contour plots depict the synchronization parameter 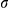 in dependence on 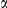 and 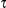 for attractive coupling (panel a) and repulsive coupling (panel b). Tongues of synchrony (white) emerge due to an intricate interplay between the inherent dynamics of each neuron constituting the scale-free network and the locking between the information transmission delay length and the oscillation period of bursting.

Merging these observation into an overall insight about delay-induced synchronization transition on scale-free networks with attractive and repulsive coupling, we show in Fig. 5 contour plots of 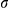, which depend on the two main parameters 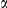 and 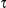 for the two types of coupling separately. The emergence of highly synchronous tongue-like regions in the two-dimensional parameter plane agrees perfectly with the reasoning we have outlined above. As the information transmission delay increases the neuronal activity enters and exits synchronous regions in an intermittent fashion. Simultaneously, as 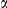 increases, the average period of bursting increases nearly linearly according to the results presented in the bottom panel of Fig. 4, thus giving an upward momentum to the white regions. However, when 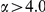 the average oscillation period drops sharply, which terminates the white “tongues” of synchrony rather abruptly and shifts the optima toward much smaller 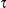. Altogether the presented results are in agreement with those presented in Figs. 2 and 3.

**Figure 6:**
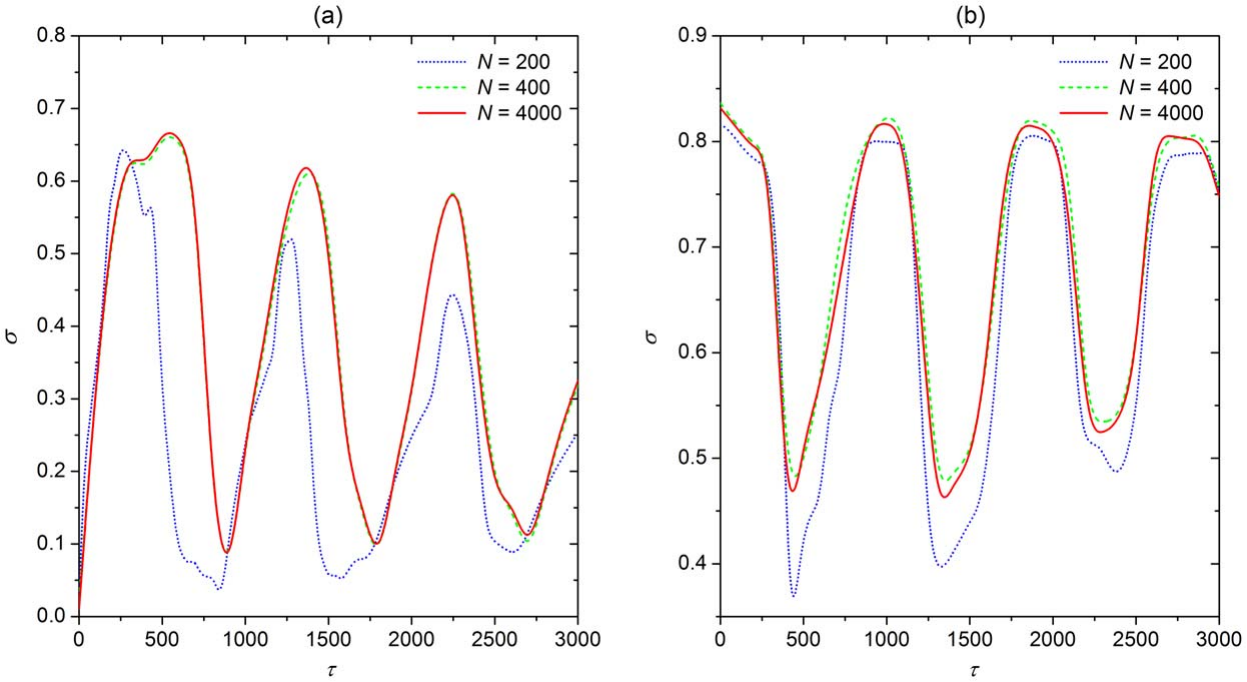
Dependence of the synchronization parameter 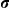 on 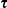 for different values of the system size 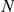. (a) Attractive coupling. (b) Repulsive coupling. Other system parameters are: 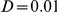, 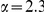. It can be observed that the results vary fairly insignificantly as the system size increases.

**Figure 7:**
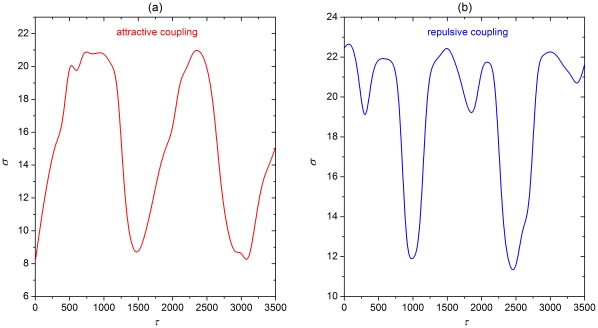
Dependence of the synchronization parameter 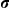 on 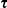 for type II neuronal dynamics. (a) Attractive coupling. (b) Repulsive coupling. Other system parameters are: 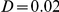, 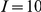 and 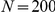. Presented results are qualitatively identical to those obtained with the Rulkov map.

**Figure 8:**
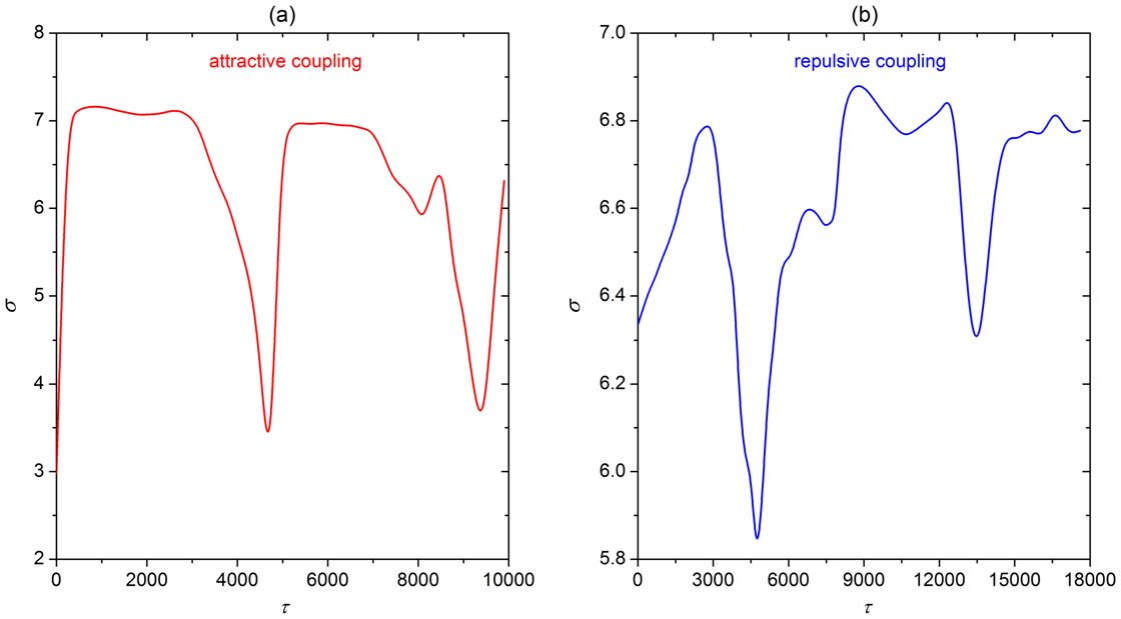
Dependence of the synchronization parameter 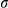 on 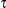 for type I neuronal dynamics. (a) Attractive coupling. (b) Repulsive coupling. Other system parameters are: 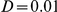, 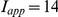 and 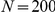. As in Fig. 7, the presented results are qualitatively identical to those obtained with the Rulkov map, thus indicating their independence on the particularities of the governing neuronal dynamics.

In what follows, in order to test the generality of the above results, we investigate the impact of different system sizes 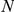 and different models of neuronal dynamics, including those of type I and type II. Firstly, for different system sizes, results depicted in Figs. 6(a) and (b) show clearly that the variations of 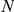 do not notably influence the outcome of our simulations. In fact, the minima of 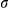 remain located at about the same values of 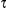 irrespective of 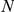. In order to validate our conclusions for different types of neuronal dynamics, we choose the famous Hodgkin-Huxley model (type II) and the Morris-Lecar model (type I) to describe the dynamics of individual network nodes (both models are given in the Methods section under “Alternative models of neuronal dynamics”). Using these two models, we investigate the synchronization transition when the delay is varied. It is shown in Figs. 7 and 8 that irrespectively of the type of the governing neuronal dynamics, intermittent synchronization transitions can still be observed for both the attractive as well as repulsive coupling when the delay is increased. More importantly, the phase locking between the delay and the period of oscillators persists in a way that is identical to what we reported above for the Rulkov model. Hence, the obtained results are also deemed robust against the variations of the neuronal dynamics.

**Figure 9:**
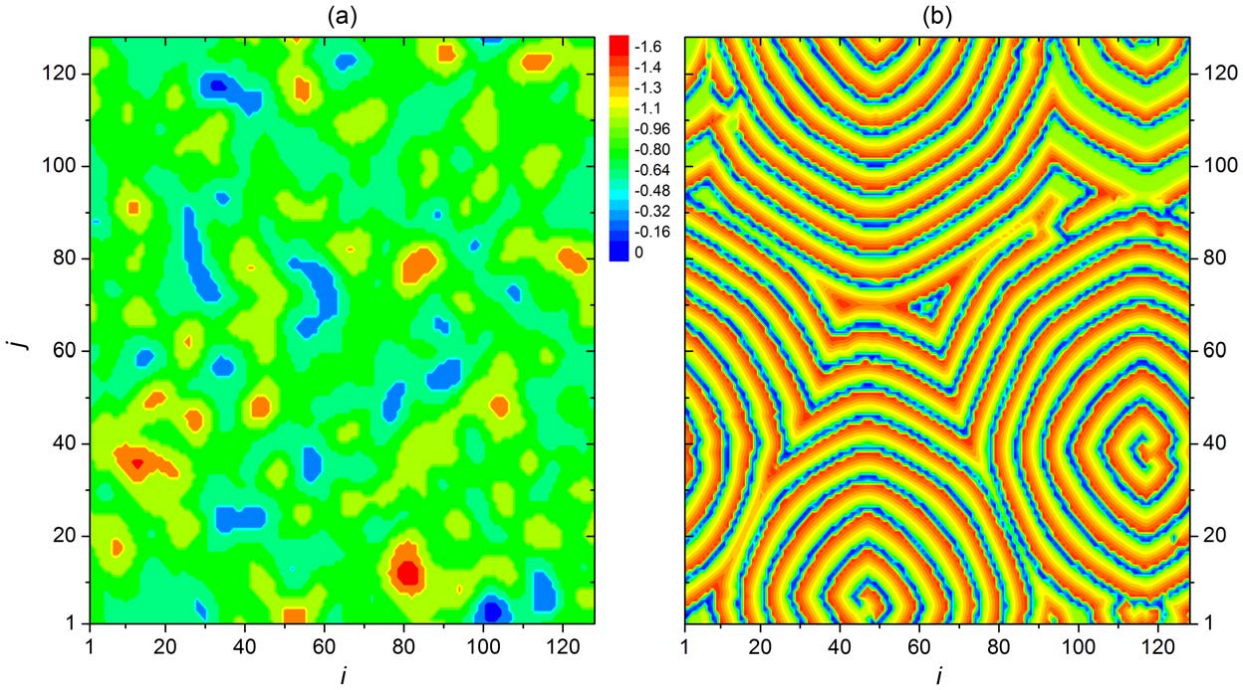
Delay-induced spatial pattern formation on the square lattice populated by diffusively coupled Rulkov neurons. Both panels depict values of 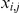 on a 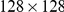 square lattice at a given (representative) discrete time 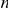. The information transmission delay 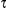 is equal to: (a) 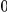, (b) 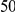. Coloring in both panels is linear, as depicted by the color strip in the middle, although the scale for the left panel was made much narrower to make the small deviations from the steady state (before it was completely reached) visible. Other system parameters are: 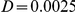, 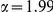. It can be observed (see panel b) that appropriate information transmission delays evoke ordered excitatory waves with a well-defined spatial frequency.

Lastly, we construct a square lattice occupying 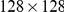 neurons, whose nodes are modeled by the Rulkov map. Here we set the parameter 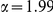, so that every neuron operates in the excitable regime. Starting with random initial conditions, the results in Fig. 9(a) evidence that as the delay equals 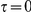, there is no pattern formation observable and each neuron approaches its excitable steady state value. On the other hand, however, Fig. 9(b) features coherent waves of excitation that appear as the delay equals 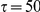, which emerge due to the locking between the delay length and the characteristic transient time of the local neuronal dynamics. Hence, it can be concluded that appropriate information transmission delays can also evoke ordered waves of excitation in the spatial domain, thus adding to their importance for the functioning of neuronal tissue.

## Discussion

We have studied delay-induced synchronization transitions on attractively and repulsively coupled scale-free neuronal networks that were locally modeled by the Rulkov map. We have shown that, irrespective of the type of couplings, information transmission delays play a pivotal role in ensuring synchronized neuronal activity. By attractive and repulsive couplings, the synchronization of bursting oscillations was found undulating intermittently as the delay was increased. However, while for attractive coupling the regions of high synchronization appeared every integer multiple of the average oscillation period, for the repulsive coupling they appeared every odd multiple of the half of the average oscillation period. Aiming to explain these observation, we have argued that by attractive coupling the intermittent outlay of synchronized regions emerges due to the locking between the delay length and the average oscillation period of bursting oscillations of individual neurons constituting the scale-free network. Conversely, by repulsive coupling the emergence of antiphase synchronization indicates locking between the delay and odd multiples of one half of the average oscillation period. Our results indicate that information transmission delays can either promote or impair synchrony among neurons and can thus effectively supplement other mechanisms of synchronization [49], [50] on scale-free networks, which arguably constitutes an important ingredient of interneuronal communication. These conclusions seem to be supported by actual biological data, stating that conduction velocities along axons connecting neurons vary from 20 to 60 m/s [51]. Real-life transmission delays are thus within the range of milliseconds, suggesting that substantially lower or higher values may be preclusive for optimal functioning of neuronal tissue. Repulsive coupling, as we have considered it in this study, is in fact an inherent ingredient of several biological systems, in particular those that contain dynamical units that are in “competition” with each other. Known examples are the inhibitory couplings is present in neuronal circuits associated with a synchronized behavior in central pattern generators or calcium oscillations in epileptic human astrocyte cultures [42]. We hope that these results will foster our understanding of the observed neuronal activity.

## Methods

The map proposed by Rulkov [52], [53] determines the dynamics of individual nodes forming the scale-free network. It captures succinctly the main dynamical features of the more complex time-continuous neuronal models, but simultaneously allows an efficient numerical treatment of large systems [54]. Accordingly, the spatiotemporal evolution of the studied network with information transmission delay is governed by the following iteration equations
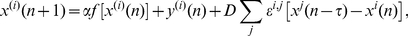
(1)

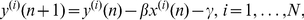
(2)where 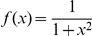 is a nonlinear function warranting the essential ingredients of neuronal dynamics, 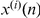 is the membrane potential of the 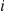-th neuron and 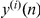 is the variation of the ion concentration, the two representing the fast and the slow variable of the map, respectively. The slow temporal evolution of 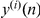 is due to the small values of the two parameters 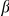 and 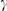 that are here both set equal to 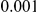. Moreover, 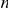 is the discrete time index, while 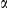 is the main bifurcation parameter determining the dynamics of individual neurons constituting the scale-free network. In [52] it was shown that for 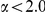 all neurons are situated in excitable steady states 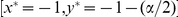, whereas if 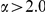 complex oscillatory and bursting patterns can emerge via a Hopf bifurcation. Importantly, we set the coupling strength equal to either 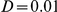, corresponding to attractive coupling, or 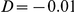, corresponding to repulsive coupling. Parameter 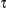 is the information transmission delay that together with 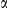 represents the two crucial parameters that are varied in the realm of this study.

As the interaction network between neurons we use the scale-free network generated via growth and preferential attachment as proposed by Barabási and Albert [55], typically consisting of 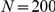 nodes or more. Each node corresponds to one neuron, whose dynamics is governed by the Rulkov map, as described above. In Eq. (1) 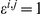 if neuron 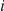 is coupled to neuron 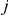 and 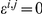 otherwise. Following [55], the preferential attachment is introduced via the probability 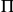, which states that a new node will be connected to node 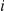 depending on its connectivity 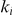 according to 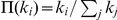. Here, 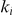 is the degree of node 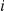 (the degree of a node is the number of links adjacent to it). This growth and preferential attachment scheme yields a network with an average degree 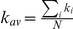, and a power-law degree distribution with the slope of the line equaling 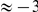 on a double-logarithmic graph. We will use Barabási-Albert scale-free networks having 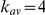 throughout this work.

In order to study synchronization transitions quantitatively, we introduce, by means of the standard deviation, a synchronization parameter 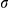 (see *e.g.* [56]), which can be calculated effectively according to:
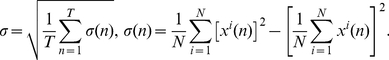
(3)


In particular, 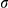 is an excellent quantity for numerically effectively measuring the spatiotemporal synchronization of excitations, hence revealing different synchronization levels and with it related transitions. From Eq. (3) it is evident that the more synchronous the neuronal network the smaller the synchronization parameter 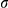. Accordingly, in the event of complete synchrony we have 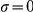. Presented results were averaged over 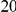 independent runs for each set of parameter values to warrant appropriate statistical accuracy with respect to the scale-free network generation and numerical simulations.

### Alternative models of neuronal dynamics

The full Hodgkin-Huxley model is given by the following equations [57]:
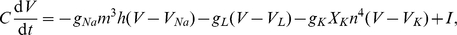


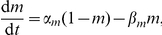


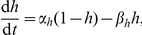


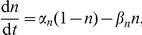
where 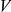 is the transmembrane potential of the neuron, and 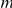, 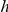 and 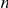 are the corresponding gating variables (probabilities) characterized by a two-state, opening or closing dynamics. The voltage-dependent opening and closing rates are given explicitly by the following expressions:
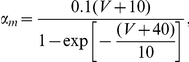


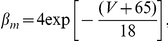


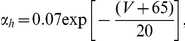


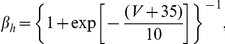


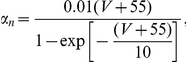


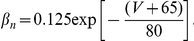



The membrane capacity 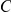 = 1 (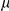
F/cm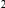), parameters 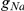, 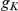 and 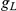 are maximal sodium, potassium and leakage conductances, 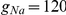 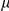
F/cm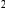, 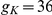 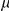
F/cm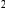 and 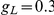 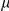
F/cm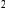, respectively, 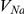, 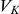, and 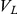 are the reversal potentials, 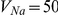 mV, 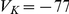 mV, 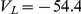 mV and 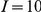 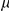.

The dynamics of the type I Morris-Lecar neuron is described by the following equations [58]:
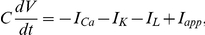


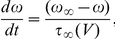


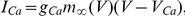


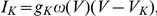


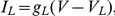
where 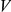 is the cell membrane potential in mV, 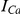 is the depolarizing calcium current, 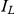 is the passive leak current, respectively, 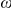 is the activation of the repolarizing potassium current 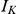, 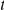 is time in ms, and 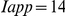 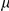 A/cm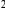 is the applied current. The remaining parameters are 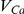 = 120 mV, 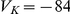 mV, 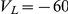 mV, 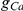 = 4 mS/cm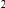, 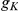 = 8 mS/cm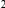, 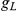 = 2 mS/cm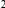. The steady state activation of the calcium current is:
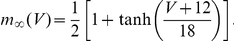



The potassium current activation amplitude and activation rate are: